# Mechanoregulation of Wound Healing and Skin Homeostasis

**DOI:** 10.1155/2016/3943481

**Published:** 2016-06-20

**Authors:** Joanna Rosińczuk, Jakub Taradaj, Robert Dymarek, Mirosław Sopel

**Affiliations:** ^1^Department of Nervous System Diseases, The Faculty of Health Science, Wrocław Medical University, Bartla 5 Street, 51-618 Wroclaw, Poland; ^2^Department of Physiotherapy Basics, Academy School of Physical Education in Katowice, Mikolowska 65 Street, 40-065 Katowice, Poland

## Abstract

Basic and clinical studies on mechanobiology of cells and tissues point to the importance of mechanical forces in the process of skin regeneration and wound healing. These studies result in the development of new therapies that use mechanical force which supports effective healing. A better understanding of mechanobiology will make it possible to develop biomaterials with appropriate physical and chemical properties used to treat poorly healing wounds. In addition, it will make it possible to design devices precisely controlling wound mechanics and to individualize a therapy depending on the type, size, and anatomical location of the wound in specific patients, which will increase the clinical efficiency of the therapy. Linking mechanobiology with the science of biomaterials and nanotechnology will enable in the near future precise interference in abnormal cell signaling responsible for the proliferation, differentiation, cell death, and restoration of the biological balance. The objective of this study is to point to the importance of mechanobiology in regeneration of skin damage and wound healing. The study describes the influence of rigidity of extracellular matrix and special restrictions on cell physiology. The study also defines how and what mechanical changes influence tissue regeneration and wound healing. The influence of mechanical signals in the process of proliferation, differentiation, and skin regeneration is tagged in the study.

## 1. Introduction

Skin is a multifaceted biological system which integrates different cells in the area of a tightly organized extracellular matrix. It is exposed to many external and endogenous factors which disintegrate its structure and functions. Skin has unique plasticity and regeneration ability. The reconstruction of anatomic continuity and restoring functions of damaged tissues is a complex, dynamic process coordinated in time referred to as wound healing. The process of wound healing is divided into four consecutive stages: homeostasis, inflammation, reepithelization, and tissue remodeling. These stages are tightly organized and precisely regulated by a complex of interaction between cells, signalization pathways, and extracellular matrix (ECM) [1, 2].

Immediately after wounding, the blood vessels close, fibrin aggregates are formed, growth factors (such as PDGF and EGF) are released, and cells associated with inflammation (monocytes, neutrophils) migrate into the wound. During the next 1–3 days, epidermal keratinocytes, almost damaged, migrate to the wound bed reproducing layer of the epidermis. This process, described as reepithelization, is crucial in regeneration of functional epidermis and prevents the development of infections. Dermal fibroblasts translocate towards the wound area and start the synthesis of ECM components and take part in ECM remodeling. Fibroblasts in the wound area transform into myofibroblasts whose contraction is responsible for tightening wound borders. In this stage, myofibroblasts strongly proliferate and synthesize components of ECM while maintaining tissue integrity and promoting its regeneration [3].

Developing a new, healthy tissue in the wounded area is dependent on cell proliferation, migration, and differentiation. Disorder of mechanisms which regulate these processes at any stadium leads to impaired wound healing. Impaired wound healing may be either slow (as in the case of diabetes, bedsores, or exposure to radiation) or accelerated (related to hypertrophy and keloid scars). In case of accelerated healing, there is a large amount of deposition of extracellular matrix, increased cell proliferation, and wound vascularization [4].

Human skin is an organ which, in an active manner, reacts to physical forces affecting it. Skin cells react to mechanical forces and their specific reaction is crucial to the way wounds behave in physical environment. The* in vitro* studies show that fibroblasts and keratinocytes are responsible for mechanical stimulation and almost all aspects of cellular behavior may be subject to modulation [5]. It becomes more obvious that improper mechanotransduction is the cause of many pathological changes, including impaired wound healing and scar formation [6].

The improper process of wounds cicatrizing, following past injury, is often the cause of many functional complications and aesthetic problems, and traditional therapies often turn out to have limited effectiveness in treatment [7]. The formation of hypertrophic scars is a major problem and therapeutic regimens as surgery, injections of corticosteroids, or radiation do not give the expected results. In contrast, high efficacy in reducing the formation of hypertrophic scars was observed in therapies based on mechanical impact on wound environment [8]. Preclinical studies have shown that excessive scarring may be limited by the equalization of mechanical forces acting on wounds and by maintaining the mechanical equilibrium of wound environment via suitable compression covers. Discharge of mechanical tensions, after surgical incisions made during abdominoplasty, limits wound scarring. Phase I trials have shown that the use of compression bandages for 8 weeks after surgery significantly affects the process of wound healing and improves the appearance of scars when compared to patients without such intervention [9].

Another form of pathological scarring is keloid changes, whose etiology is quite poorly known. The reasons for pathogens include genetic disorders, apoptosis, dysregulation of mesenchymal-epithelial signaling, and variations of mechanical tension in wound environment [10]. As a result of the mechanical tension fibroblasts which form keloid scars exhibit a higher expression of profibrotic cytokines and increased collagen synthesis in response to activation of focal adhesion kinase (FAK). Mechanical stress and mechanical stimulation in wounds healing clearly increase the likelihood of developing keloids, and controlled mechanomodulation therapies can limit the progression of these changes [11].

## 2. Mechanical Regulated Cell Proliferation and Differentiation

A widely accepted concept of homeostasis describes that structural damage to tissues activates the response of the organism to restore impaired mechanical equilibrium of the skin. Wounds of different etiologies and anatomic location have specific mechanical properties that affect the way of their healing [12].

Mechanotransduction enables a cell to sensing and to quickly adapting to mechanical forces and physical limitations. The way in which a cell receives mechanical stimuli, how such mechanical signals are transmitted into cells, and how those signals regulate gene expression and protein synthesis are important issues. Cell structures described as mechanosensors are a cell membrane [13], mechanosensitive ion channels [14], glycocalyx [15], focal adhesions, and proteins and intercellular complexes [16].

The essential mechanism of mechanotransduction is based on converting mechanical signal received by cell structures into intercellular signaling pathway which determine its interaction with different cofactors and target gene specificity [17]. These mechanical stimulations are then targeted into signaling pathway induced by soluble factors and consequently regulate transcriptional changes. In an alternative model, the cell itself is considered a compartmentalized mechanical body with given physical properties such as its viscosity, elasticity, or stiffness [18].

Cells are influenced by mechanical forces suitable for their environment, such as softness or rigidity of extracellular matrix (ECM), differentiated adhesion to substrate, or the tension exerted by neighboring cells. The physical properties of ECM influence micro- and nanotopography of integrins which bind ECM components. Integrins are a part of protein complexes which form focal adhesion. They are responsible for producing a direct physical connection between the components of the extracellular matrix (e.g., collagen, laminin, fibronectin, and vitronectin) and the adapter proteins of actin cytoskeleton. The process of binding of actin filaments with the participation of integrins generates tensions in cells, which, at the same time, activates actin associated proteins that regulate polymerization of F-actin. This affects the spatial organization of actin filaments and integrins and thereby increases cell adhesion to the ECM [19].

The interaction of actin filaments and myosin is responsible for the contractile force and ultimately the creation of intracellular mechanical tension. The structure and dynamics of formation of actin-myosin complexes are regulated by Rho family GTPases.

Inhibition of Rho, ROCK, and MYLK (myosin light chain kinase) or the inhibition of the polymerization of actin filaments reduces the strength of internal tension of a cell and causes a change in cell shape. A similar effect can be achieved when culturing cells on a soft substrate [20]. This effect is closely related to cell shape and intracellular tension forces [21].

The cells adjacent with large adhesive area to the substrate have ability to proliferate in more intensive manner, whereas the cells adjacent with a small adhesive area do not proliferate and most often die [22]. Preventing cells from spreading on the substrate leads to shifts in the organization of actin filaments and activation of transcription factor SRF (Serum Response Factor), which together with cofactor MAL is transported to the nucleus. SRF-MAL complex activates the transcription of c-FOS and JUNB elements of AP-1 complex [23]. Activation of the SRF is the result of a shift in cell shape; it is independent of the density and composition of the extracellular matrix and the assembly of focal adhesives [22]. As SRF is a downstream effecter of Rho-A and actin polymerization, it provides an important link between the cytoskeleton and gene regulation. A direct connection between SRF and various cellular responses to mechanical and biophysical stimuli has been demonstrated. In keratinocytes, myocardin-related transcription factor-A (MRTF-A) and SRF are required for shape induced terminal differentiation, whereas, in fibroblasts, the T-cell factors family (TCF) of cofactors control the switch from proliferation to transcription following loss of adhesion [24].

Microenvironmental signals determine the fate of epidermal stem cells: the loss of contact with the basement membrane and the transfer of cells to a higher layer direct them to terminal differentiation. Keratinocytes cultured* in vitro* in suspension exit cell cycle and enter the differentiation pathway, regardless of binding ligands with appropriate integrins. The direction of differentiation of mesenchymal stem cells (MSCs) also determines their geometry. In the* in vitro* environment, the outspread MSCs cells, strongly adherent to the substrate, differentiate into osteoblasts, and when in culture they take on a spherical shape; they differentiate into adipocytes. Equally important determinant of differentiation of mesenchymal stem cells is the rigidity of their immediate surroundings. MSCs differentiate into osteoblasts when grown on synthetic substrate of bone-like rigidity, into myoblasts when they grow on a substrate of intermediate rigidity, and into neurons and adipocytes on a soft substrate. Cell environment, like growth factors, may affect the growth of the cell population and the direction of differentiation [19, 25].

The fate of epidermal stem cells and mesenchymal cells appears to be regulated by mechanical feedback from the extracellular matrix. Stem cells put tension on the extracellular matrix and receive in return the force of environmental influences which determines their fate. ECM with open weave does not provide stem cells with an appropriate signal, because of which it is not able to respond while fitting focal adhesion and initiating signaling to MAPK/ERK pathway. The strength of binding with extracellular matrix proteins and the density of integrins affect the shape and direction of keratinocyte differentiation [22].

It has been shown that the cytokeratin cytoskeleton has a significant impact on the mechanics of keratinocytes and signals mechanotransduction. Hemidesmosomes, like focal adhesion, also receive the mechanical forces of the extracellular matrix in the epidermis [26]. Human keratinocytes, exposed to mechanical stress by cell stretching, activate signaling pathways dependent on calcium ions which regulate cell proliferation and differentiation [27].

Extracellular matrix in an injured tissue changes its chemical composition and stiffness, which initiates the repair process. Mechanical signals received by fibroblasts guide their transformation into myofibroblasts [28]. In a freshly damaged tissue, extracellular matrix is soft and rich in fibrin. Fibroblasts grown on the soft substrate in 3D cultures have little adhesion and poorly developed stress fibers [29]. In contrast, when cultured on a hard substrate, they form a number of focal adhesion and stress fibers, although they do not yet show the presence of myofibroblasts markers such as *α*-smooth muscle actin (*α*-SM actin). The transformation of myofibroblasts occurs in cultures, in a rigid 3D collagen matrix, or during wound granulation and fibrotic tissues, but, nevertheless in all cases, it is necessary to stimulate with TGF-*β*1 [30].

In the process of wound healing, the contraction of the myofibroblasts increases the rigidity and mechanical tension of the extracellular matrix. In turn, the increase of ECM rigidity and mechanical signals, generated on the basis of positive feedback, further stimulates the differentiation of myofibroblasts. The role of the mechanical tension in the stimulation of the activity and differentiation of myofibroblasts was demonstrated experimentally on skin wounds in mice. The wounds were subjected to mechanical tensions by stretching and splinting, and the increasing activity of myofibroblasts showed the intensification of scarring, to a large measure resembling the human hypertrophic scars. Declining mechanical stress or the decrease of the rigidity of extracellular matrix can induce apoptosis and decrease the expression of *α*-SM actin and myofibroblasts' ability of contraction [31].

Mechanical signaling, received by integrins in a direct way, activates transcription of *α*-SM actin genes [32]. In response to mechanical signals and stimulation of TGF-*β*1 in myofibroblasts, the expression of genes responsible for the synthesis of collagen and extracellular matrix proteins increases [33], which results in changing of mechanical properties of the injured tissue. In the wound site, the TGF-*β*1 is released (from alluvial cells of connective tissue, platelets, and myofibroblasts) in a form which is inactive in a complex with LAP (latency-associated peptide). TGF-*β*1/LAP complex is bound to extracellular matrix proteins forming a reservoir of latent form of TGF-*β*1. Myofibroblasts, by receiving the tension forces from the extracellular matrix, express integrins which bind LAP, which activates TGF-*β*1 and enables binding with cell membrane receptors [31]. Both the increase in the mechanical tension and the contraction of the myofibroblasts can activate cells by increasing their contraction ability and their synthetic activity of extracellular matrix components. On the other hand, blocking integrins (*α*v*β*5, *α*3*β*1, *α*11*β*1, and *α*v*β*1) involved in the activation of latent forms of TGF-*β*1 is an alternative pathway of regulating myofibroblasts' activities in the process of wound healing [34].

A collective, coordinated cell migration is an important part in the mechanism of wound healing [35], similarly as in embryonic development [36], and invasion of tumor cells [37]. In model systems of* in vitro*, the epithelial cells (MDCK), released from the microtemplates, distinguish a group of cells leaders which initiate migration and generate traction forces towards migration [38]. Adhesive junctions maintain tissue consistency, generate tension between cells, and entail the entire group of cells. The rigidity of the substrate, in return, regulates the migration of cells through the activation of myosin II [39]. Cell response to the rigidity of the substrate also depends on the specific integrins: for example, in myoepithelial cells, the differences in the kinetics of binding between the *α*5*β*1 integrin and *α*v*β*6 determine the level of the traction force which the cell can generate in response to a predetermined rigidity of the substrate. Overexpression of these integrins' receptors was described during skin wound healing, which may point to their important mechanoreceptive role in the regulation of normal tissue repair [40].

In comparison with other epithelia, keratinocytes generate particularly strong intercellular connection that enables the collective migration occurring even in an environment with little or highly dispersed ECM substrates for specific integrin receptors [41]. Junction between cells can promote wound reepithelization with limited or varied adhesion to ECM and facilitate wound closure in the absence of specific integrins. Therefore, a key area of research that will allow describing the precise mechanism involved in slow or accelerated wound healing is to investigate the expression of specific integrins, components of the cytoskeleton, and target proteins involved in mechanotransduction, as well as conduct a thorough analysis of mechanical wound environment [42].

The properties of extracellular matrix determine cell shape, thanks to which they can influence the cell cycle regardless of intercellular regulative mechanisms. The process of when cells move through to the phase of S cycle depends on critical size, shape, and also the reception of mechanical signals which indicate spacial limitations of a cell in its environment. This mechanism is related to a single cell and cell population which compose a tissue, and it results in stopping cell crowding, exclusion from tissue, and apoptosis [43].

In the* in vitro* models of cell kinetics, it was demonstrated that the elimination of spatial limitation causes cells to quickly move through to the phase S cycle. This mechanism is activated when the tension of the actin gets down. The degree of cytoskeleton tension is a mechanical signal that determines the spatial size of a cell and that may be subject to further transduction through pathways which regulate cell proliferation, such as yes associated protein (YAP), S-phase kinase associated protein 2 (SKT2), or extracellular signal-regulated kinases (ERK) [44, 45]. Changes of the dynamics of the cytoskeleton, in response to changes in the mechanical environment of the cells, extend in a short period of time (faster than the average cell cycle time). Thus, open space can increase cell proliferation, but also the short duration of signal may limit the overregulation of proliferation. Functioning of this system has been demonstrated* in vivo* in processes related to the development and organogenesis and in pathological conditions associated with abnormal proliferation [46].

This mechanism, in a simple manner, may be observed in the model of cell regeneration and regulation of cell proliferation during wound healing. Cells do not need to register information related to wound size; they only invade the space which is available at that particular moment. Lowering mechanical tension of cells activates the process of proliferation and migration. Further cell divisions fill in the open space and they project the primal state of spatial limitation in* de novo* tissue which is being formed. In consequence, cells shrink back to the size they have in an inactive state and their further divisions are inhibited. In conclusion, it may be stated that in the processes of development, regeneration, and cancer cell invasion, the progression of cell cycle on the border of G1-S-phases may be regulated by mechanosensitive control spots which receive spatial limitations of cells environment [47].

Understanding the process of mechanotransduction requires also an answer to a question in what manner mechanical forces are transmitted to the cell nucleus. There are proofs that the direct mechanical coupling of a cytoskeleton, plus changes in the nuclear envelop and in nucleoskeleton, may be an alternative or additional way of regulation of genes expression [48]. Changes in localization or structure of the cell nucleus are observable in many processes, such as cell division, migration, and differentiation. To keep a localization of the cell nucleus appropriate for the cell type is an active process, dependent on physical connection between the cytoskeleton and the structure of the cell nucleus [49].

Recently, the proteins are responsible for these connections we discovered; they complete the complex called LINC (Linker of Nucleoskeleton and Cytoskeleton). This complex is composed of SUN proteins (Sad1p, UNC-84) and KASH proteins (Klarsicht/ANC-1/Syne Homology). SUN proteins and KASH proteins bind with perinuclear space forming a bridge which connects cytoskeleton with nucleoskeleton. This bondage plays the main role in many cell processes and is responsible for maintaining correct position of the nucleus in a cell. The LINC complex participates in cells migration and intercellular transportation dependent on both microtubules and actin filaments. The disintegration of LINC complex results in disorganization of actin skeleton and disturbs the mechanics of a cell [50, 51]. The LINC complex transmits not only the tension generated by the cytoskeleton to cell nucleus, but also the mechanical tension directed at cell surface. The mechanical tension, which is received by cell surface adhesion receptors, influences the structure of nuclear envelope. These observations prove that mechanical stress may be transmitted from extracellular matrix to the cell nucleus. Mechanical cell stimulations through stretching or compression influence the shape of the nucleus and the organization of nucleoplasmatic structures [52].

The way in which the transmitted mechanical forces influence genes expression is an important issue. The mutation of the emerin gene to its phosphoresistant form influences the transcription profile of genes dependent on serum response transcription factor (SRF), which shows that mechanical forces received by nucleus influence genes expression [53]. This conception is proved by latest studies which show that lamins and emerin regulate translocation to MLK1 nucleus and transcription of genes dependent on SRF [54]. Further studies show that mutations of A-C laminae influence the reception and transduction of mechanical signals on the YAP dependent pathway [55]. It was also stated that A-C lamins level is regulated in response to shifts in the extracellular matrix rigidity and is related to dephosphorylation and stabilization of lamins [48]. The decrease of phosphorylation of lamins may result from the processes of regulation of specific nuclear kinases or phosphatases dependent on mechanical tension. The study has shown that phosphorylation of emerin, in response to mechanical tension, strengthens connections between A-C laminae and LINC complex. Lamina and emerin in response to mechanical stress begin to interact with chromatin modifying its structure, because of which they influence genes expression [56]. In the studies related to isolation of the cell nucleus, it was observed that lamina A is subject to conformational shifts in reaction to mechanical stress. These observations prove that nuclear proteins receive and participate in the transduction of mechanical signal and bind with biochemical signalization which regulates the activities of nucleoskeleton proteins. Transferring the mechanical force onto LINC complex may cause conformational shifts of emerin and its phosphorylation regardless of the activity of specific kinases [56]. A similar mechanism of mechanotransduction was described for focal adhesion protein p130-Cas [57].

## 3. Transcription Factors Regulated by Mechanical Forces

### 3.1. Transcriptional Coactivators YAP and TAZ

YAP (yes associated protein) and its homologue TAZ (transcriptional coactivator with transcriptional coactivator with PDZ binding motif) are the key effectors of the Hippo signaling pathway. Their activities are inhibited by the main kinases of Hippo pathway, LATS 1/2, on the way to phosphorylation [58]. YAP and TAZ activate genes transcription through interaction with transcription factors belonging to a TEAD family (TEA domain family member) [59]. The genes induced by YAP/TAZ are involved in the regulation of cell proliferation, apoptosis avoidance, amplification of stem cells, control of the size of organs, and tissue regeneration [60].

YAP/TAZ transcription coactivators, besides being involved in signal transmission of the Hippo pathway, interact with other proteins and respond to mechanical stimulation of a cell. Many proteins which bind with YAP and TAZ molecules have the ability to bind actin and can regulate, or be regulated by, changes in the structure of the cytoskeleton [44, 61].

Many studies show that mechanical signals regulate the activities of YAP and TAZ through the pathway, which may act simultaneously with a classic cascade of Hippo pathway kinases. YAP and TAZ molecules are inactivated when F-actin is depolymerized or when the activity of Rho-GTPases is inhibited. The knockout of LATS1 and LATS2 does not rescue the activity of YAP and TAZ in the presence of actin polymerization inhibitor or in cells cultured on soft hydrogel surface. In addition, under the same conditions, the activity of mutant TAZ susceptible to LATS is permanently inhibited. The cells which grow in suspension (without contact with the components of the extracellular matrix) show no YAZ activity and undergo anoikis. The LATS knockout only partially rescues cells from death [44, 62]. It can be assumed that a spread of F-actin cells prevents the action of unknown factors inhibiting YAP and TAZ in a manner largely independent of LATS1/2.

Subcellular location and activity of YAP/TAZ are regulated by the rigidity and topography of cell substrate and remodeling of cytoskeleton [63]. The change in cell shape, into more flat and related shifts in the organization of cytoskeleton in response to integrin signaling, is an important factor which keeps YAP/TAZ active. YAP and TAZ coactivators in cells cultured on a stiff substrate are located in cell nucleus and transcript actively. However, when cells are transferred onto a soft substrate, they are removed from nucleus and thereby become functionally inactive. Similar regulation of activities of YAP and TAZ was shown in cells which grow on a micropatterned substrate. In such culture conditions, cells differ in terms of the degree of spread-out in such a way that the cells which are placed on large fibronectin islands, which enables their spread-out, have active YAP/TAZ with nuclear location. However, when cells are placed on small adhesive islands, the cytoplasmic form of YAP/TAZ is inactive. Cells with YAP/TAZ knockout proliferate extensively on high adhesive or rigid substrate, and they have a phenotype typical for cells (without knockout) cultured on a weakly adhesive or soft substrate. Activation of YAP/TAZ signaling on stiff substrates involves actomyosin-driven cytoskeletal tension but is independent of the Hippo pathway components LATS and MST kinases [44]. Although cell-cell adhesion stimulates the Hippo pathway and inhibits YAP/TAZ, mechanical signals from the ECM can override Hippo pathway signalizations [45]. However, it remains to be precisely determined how actomyosin tension has direct effects on YAP/TAZ activity [61, 64].

Artificially forced changes in cell shape through their flattening, in a manner which does not involve integrins (with polylysine as substrate), maintain nuclear location of YAP. Moreover, inhibiting the activities of focal adhesives components, such as FAK or SRC kinases, does not affect YAP/TAZ activities. The presence of large amounts of G-actins (monomers) in cytoplasm does not affect YAP/TAZ activities. The information presented shows that an important factor which maintains activity of YAP and TAZ is the change in cell shape to be more flat and all shifts in cytoskeleton organization related. It seems that the activity of YAP and TAZ is dependent on the organization of actin filaments which are organized into stress fibers, or which form bundles of shrinkable networks that enrich cells of outspread shape [44, 45].

The specific structure of the F-actin may, in a physical manner, sequester inhibitory molecules or may provide a platform which enables their posttranslating modifications which block interactions with YAP and TAZ. Cells cultured on a soft substrate with limited surface of contact remodel actin skeleton in such a way that the inhibiting factors may be released or activated. There is also a possibility that actin severing proteins regulate the activity of YAP/TAZ, exposing or covering appropriate sites that bind regulative proteins, by controlling the ability of interaction directly with YAP or TAZ or through their partners. Moreover, flattening shape of cells and consequential reorganization of actin skeleton may promote the activation of positive cofactors that enable translocation of YAP and TAZ to cell nucleus and their activation [45, 62, 64].

The activity of YAP and TAZ is also regulated by a cell-cell contact and by formation of new intercellular contacts. Many positive and negative YAP and TAZ regulators are adherens junction proteins and tight junction proteins. Angiomotins (AMOT) and zonula occludens proteins 2 (ZO-2) interact with YAP/TAZ regulating their activity [65, 66]. The component of adherens junction protein *α*-catenin, which binds adhesive complex with actin cytoskeleton, regulates YAP activity through binding a phosphorylated complex YAP-protein 14-3-3 with the connection which binds epidermal cells [67, 68]. The next protein which is responsible for cell connections and which interacts with YAP/TAZ is zonula occludens protein 2 (ZO-2). This protein enables translocation of YAP/TAZ between the complex of tight junctions and cytoplasm and between cytoplasm and the cell nucleus. The discovery of YAP/TAZ/ZO-2 complex is extremely interesting because of the fact that proteins of tight junctions may directly affect the transcription activity of YAP/TAZ [64, 66, 69, 70].

**Figure 1:**
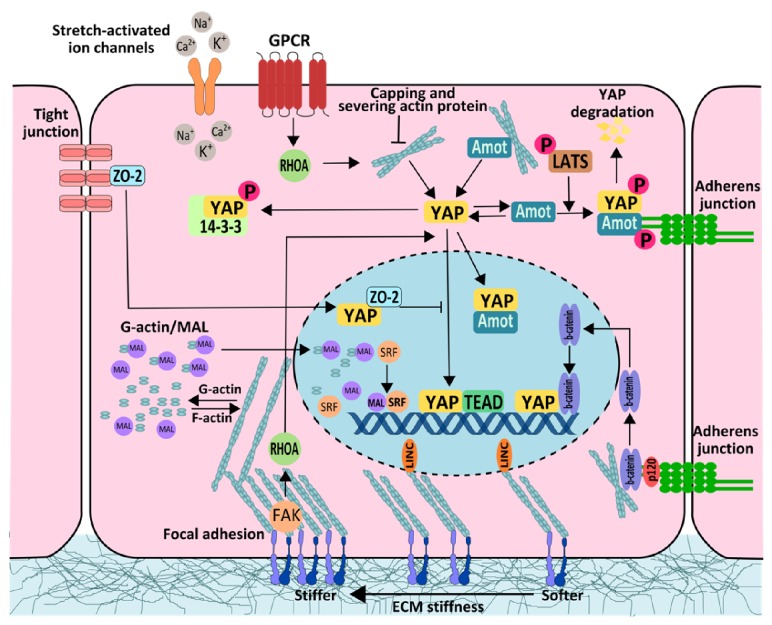
Mechanotransduction in transcriptional regulation.* Description*. Interacting with G-actin inactivates MAL. F-actin polymerization uses up amounts of unpolymerized F-actin and removes the inhibition of MAL through G-actin and released MAL binds to the SRF. This activated SRF binds to DNA and induces transcription. YAP can be inhibited by mechanisms not connected with kinases such as AMOT. AMOT protein binds actin filaments and allows YAP to enter the nucleus. If it comes to F-actin depolymerization, AMOT dissociates from actin and retains YAP in the cytoplasm. When YAP is phosphorylated by LATS, AMOT recruits ubiquitin ligase to AMOT/YAP complex and initiates the YAP proteasome degradation. The protein bound to tight junction ZO-2, together with YAP, enters the nucleus where it inhibits the activity of YAP. The p130 isoform of AMOT acts in the opposite manner and promotes nuclear localization of YAP and acts as a transcriptional cofactor of the YAP-TEAD complex. Rho GTPases control YAP/TAZ activity through canonical GPCR-linked (G-protein coupled receptors) manner or noncanonical activation of YAP through focal adhesion signaling and FAK kinase. It is hypothesized that the presence of F-actin and stress fiber formation (stress fibers) is crucial for the activation of YAP and TAZ. Upon translocation to the nucleus, they associate with TEAD transcription factor which drives transcription of proliferative genes. Rho GTPases and actin associated proteins (CAP-Z Cofilin, Gelsolin) can have a stabilizing effect on the network of actin filaments and directly or indirectly regulate YAP/TAZ translocation to the nucleus. Mechanical forces generated by ECM can be directly transmitted by the cytoskeleton to the nucleus through LINC complex. Mechanical signal transduction is received by nucleoskeleton proteins (laminae, emerin) that directly or indirectly may affect gene expression. Activation of *β*-catenin and translocation to the nucleus in response to compressive forces. *β*-catenin is structural component of adherens junctions in epithelial cells, regulating cell-cell interactions. Shuttling of *β*-catenin between the cytoplasm and nucleus is a key step in this signaling pathway. Unphosphorylated *β*-catenin can enter the nucleus and activate transcription, despite activation canonical Wnt pathway (adapted from Low et al. [74]).

AMOT as a partner which signals YAP/TAZ is particularly interesting because it interacts with actin filaments and adherens junction proteins [71]. AMOT in a cytoplasmic location promotes YAP phosphorylation and stops in cytoplasm by forming stable YAP/TAZ complexes. In effect, the transcription of target genes of connective tissue growth factor (CTGF) and cysteine rich protein 61 (Cyr61) is inhibited [65]. What is crucial, similarly to YAP/TAZ, AMOT is phosphorylated by LATS kinases, which leads to protein dissociation from actin complex, YAP suppression through 14-3-3 enrolment, ubiquitination, degradation, and inhibition of cellular proliferation [72, 73]. In a situation when AMOT is connected with actin filaments, YAP/TAZ may be directed to the cell nucleus and take active part in transcription. AMOT plays an important role in maintaining the status of F-actin and it participates in a competency bondage of YAP/TAZ with actin filaments. The increase of polymerization of F-actin results in a lowered degree of AMOT, which leads to the release of YAP/TAZ and its translocation to the cell nucleus and activation of genes dependent on the YAP-TEAD complex, and cell proliferation (Figure 1) [74].

There are some conflicting data, which shows models for the negative regulation of YAP/TAZ activity by the Angiomotins, which suggest a number of possibilities leading to localization of YAP/TAZ to the cytoplasm/cell junctions. It is noteworthy that a major mechanism of YAP/TAZ regulation is through exclusion from the nucleus and it was identified as a nuclear function for Amot-p130 in regulating YAP activity. Also, it was found that Amot-p130 is required for YAP function both* in vivo* and* in vitro*, which is contrasting to a YAP-inhibitory role for Angiomotins [75].

### 3.2. YAP and TAZ in Skin Homeostasis and Wound Healing

The process of stratification of the epidermis can be interpreted as cell differentiation induced by the loss of cell-extracellular matrix contact. The location and activation of YAP in the epidermis also appear to be dependent on the interaction of cell-extracellular matrix. In proliferating cells of the epidermis layer, YAP is localized in the nuclei, and differentiating cells of the upper layer, it is present in the cytoplasm. The increase of YAP in nuclei increases the proliferation and inhibits cell differentiation. By contrast, inactivation of YAP leads to inhibition of proliferation and premature differentiation [76].

Overexpression of YAP active form in the basic layer of the epidermis of mice embryos leads to increased proliferation of keratinocytes, impaired stratification and hyperplasia of the epidermis, and inhibition of terminal differentiation. The lack of YAP expressions causes visible decrease of proliferation, decrease of stem cells, impaired stratification, and, in effect, decrease of epidermal thickness. A similar phenotype is observable in YAP knockout or the presence of mutated protein isoforms that are unable to interact with TEAD. Activation of MST1/2 kinases causes the inhibition of YAP activity and in effect the decrease of cell proliferation and initiates their differentiation. However, in the lines of human keratinocytes HaCaT, the inactivating of MST1/2 does not affect the shift of cell phenotype. Also the LATS1/2 knockout does not affect phosphorylation and YAP activity. This indicates the alterative, independent of Hippo pathway, way of inhibiting of YAP activity in human keratinocytes [67, 76, 77].

The homeostasis of epidermis depends also on cell-cell contact. The decrease of expression of *α*-catenin in epidermis leads to the increase of YAP activities; however this dependence is especially limited for the basal layer cells. In case of a correct cell-cell contact needed to keep YAP active, the presence of other signals, which can include the shift of cytoskeleton dynamics in response to mechanical signals, is necessary [68].

In many studies carried out* in vivo* and* in vitro*, the crucial dependence between YAP and TAZ expression and a process of wound healing and tissue regeneration has been shown. The nuclear localization of YAP and TAZ has a main influence on the tissue regenerative abilities and feedback to outer signals during wound healing. During wound healing, the translocation to the nucleus and YAP/TAZ activation have a strong pleiotropic effect characterized by wound closing, cell proliferation, and collagen synthesis [78]. Knockdown of YAP/TAZ inhibits cutaneous wound healing, suggesting an important role for these factors in tissue regeneration as well. On the basis of the diverse functions of YAP/TAZ as regulators of mechanotransduction in other cell types of the skin, probably this signaling pathway also mediates epidermal mechanosensing [24].

In normal skin, YAP is located in cell nuclei of keratinocytes of the basal layers of the epidermis and hair follicular epithelium. In healing wounds, the YAP expression highly increases within the whole area of healing tissue. What is important, the nuclear localization of YAP is visible in healing areas of the wound; however, its lack is observable in normal tissue neighboring with the wound [79]. The described topography of YAP localization suggests that in a healing skin there is the activation of signaling which participates in YAP translocation to the cell nucleus, most probably as the effect of inhibition of Hippo pathway [77, 80]. Another explanation for shifts in YAP localization may be the fact of tissue loss and changing of mechanical environment in cell area, which may also activate YAP [67]. Immunocytochemical localization of TAZ in a normal tissue shows its presence mainly in the cytoplasm of fibroblasts. During intense wound healing, TAZ, in the majority of cells, relocates from cytoplasm to the cell nucleus. In addition, the expression of TAZ is stronger in the area of tissue which undergoes intensive regeneration [79].

Silencing YAP and TAZ on a mice model of wound healing with the use of appropriate siRNA leads to delays in wound closing in comparison to the control group. The effect of silencing YAP/TAZ in the wound area is associated with inhibition of cells proliferation and a decrease of collagen synthesis. Although silencing YAP/TAZ by siRNA is not a long term process, the effect of this process is clearly visible [79]. In* in vitro* conditions and in the studies on mice model of wound healing, it has been shown that silencing YAP or TAZ in fibroblasts causes a noticeable decrease level of a transforming growth factor beta 1 (TGF-*β*1), as a result of impairment of the sequence of processes related to appropriate wound healing. TGF-*β*1 is a key mediator in the process of wound healing. It is responsible for the transformation of fibroblasts to myofibroblasts, as in consequence their contraction and moving towards the wound border, for differentiation of vascular smooth muscles, and is the main simulator of collagen synthesis [81].

One of the target genes of transcriptional coactivator YAP is the gene of CTGF [59]. CTGF supports the signaling of TGF-*β*1/SMAD by suppression of SMAD-7 protein. This protein exhibits minimal expression in a healthy skin, and its level significantly increases in wounded skin [82]. In addition, the interaction of TAZ and TEAD is crucial to activation of the transcription of cysteine rich angiogenic inducer 61 gene (CYR61) [83]. CYR61 is a secreted, extracellular matrix- (ECM-) associated signaling protein and participates in the regulation a broad range of cellular activities, including cell adhesion, migration, proliferation, differentiation, and apoptosis, through interaction with integrin receptors, and is intensely synthetized by myofibroblasts during the process of wound granulation.

It has been shown that YAP and TAZ in a nuclear localization (which is dependent on the density of cells) participate in the translocation, which is induced by TGF, of SMAD to the nucleus. In contrast, when YAP/TAZ are in the cytoplasmic localization they have an inhibiting impact on the same process [84]. TGF-*β*/SMAD signaling may be also responsible for the induction of the expression of CTGF [85]. It may be assumed that, during the process of wound healing, TAZ, which directly influences the activation of TGF-*β* pathway, participates, in an indirect manner, in the process of control of the level and activity of SMAD-2 [86]. It seems that TAZ controls TGF-*β*1 signaling more effectively than YAP. TAZ knockout in a healing wound clearly reduces the transcription of SMAD-2 which is induced by TGF*β*. However, YAP which influences the activity of SMAD-7 may participate in muting TGF-*β*1 signaling [87]. SMAD-3 and SMAD-5 interact directly with and are phosphorylated by activated TGF-*β*1 receptors; also SMAD-6 and SMAD-7 bind activated TGF-*β*1, thereby preventing phosphorylation of R-SMADs [88, 89]. These observations suggest that YAP/TAZ participate in the modulation of wound healing through the mobilization of synthesis and activation of TGF-*β*1, and signals related to TGF-*β*1 induce the activities of such factors as SMAD and CTGF. Binding the signaling pathways YAP/TAZ with TGF-*β*1/SMAD is an important factor which regulates a multistage process of wound healing.

### 3.3. Serum Response Factor (SRF)

Serum response factor (SRF) is a transcription factor which was discovered in fibroblasts treated with blood serum. The cells receive sudden exposure to serum as a signal which signifies injury and tissue damage, and in consequence, they start the healing process. Serum exposure does not only result in mitogenic activation; it is a more complex process which involves the effects on fibroblasts of epithelial and endothelial cells [90].

SRF is responsible for expression of genes which regulate proliferation and cell differentiation [91]. SRF is activated by mitogenic protein kinases or Rho-A pathway and it produces a fast transcriptional response through regulation of factors, signaling proteins, and cytoskeleton components [92].

There are approximately 300 known genes which consist of SRF response elements; among them, there are genes of early cellular response (e.g., c-FOS, cyr61), whose expression is important in the process of wound healing [93]. SRF is a key factor which induces the differentiation of fibroblasts during wound healing. Overexpression of SRF, in stem cells, epithelial cells, as well as, in fibroblasts, promotes their transformation to myofibroblasts [94].

The activity of SRF may be regulated by many independent pathways. In relaxed smooth muscle cells, the relationship of SMAD-7 (inhibitor of TGF-*β* signaling) with the activity of SRF has been shown. The increase of TGF-*β* level weakens this interaction [95]. It has also been shown that the activation of integrin-linked kinases (ILK) and the SRF phosphorylation is linked to TGF-*β*1 signaling [96]. Moreover, phosphorylation at serine-103 promotes the ability to link SRF to *α*-actin of smooth muscles in proportion to the ILK activity, and it also increases protein stability. However, the ILK inactivation decreases the half-length of SRF [97].

It has been proved that one of the factors which affect SRF activity is the dynamics of cytoskeleton and the activity of cofactor G-actin MAL (Megakaryoblastic Leukaemia 1) [98]. The monomeric actin (G-actin) while binding with MAL closes proteins pathway to the nucleus and its functional inhibition. As a result of serum stimulation and Rho activation, the polymerization of actin increases and the level of actin monomers decreases. In such conditions, MAL is released to the nucleus where it starts the transcription of independent genes. Other studies show that the activity of SRF-MAL may be also regulated by forces connected with cell migration. It has been shown that the interaction between MAL and G-actin in cellular nucleus blocks MAL binding with SRF. The factors which induce polymerization of F-actin decrease the pool of free G-actin and, at the same time, they increase the availability of MAL, which may lead to SRF activation [99].

The model of* in vitro* differentiation of epidermal stem cells by mechanotransduction, with the use of microsamples of matrix islands, showed that cells growing on the larger islands form a dense network of actin filaments and stress fibers thereby reduce the pool of free G-actin and increase the availability of MAL. Availability of MAL induces the activation of SRF and genes dependent on it, such as JUNB or FOS. SRF activation through mechanical signals is an alternative or parallel way to the activity of growth factors on epidermal cells [23].

### 3.4. SKP2 (S-Phase Kinase Associated Protein 2)

SKP2 protein (S-phase kinase associated protein 2) for the first time was identified as an important element of Cyclin A-CDK kinase/S-phase complex [100]. In further studies, it was described as a protein which is bound to SKP1, with which it composes a ligase ubiquitin type SCF complex (Skp1-cullin-F-box) that takes part in the regulation of cell cycle through proteolysis dependent on ubiquitin. The SKP2 is a recognizing subunit of SCF complex and substrate is p27 protein. Ubiquitination and the proteasome degradation of p27 enable the transition S-phase cell cycle and promote cell proliferation [101].

SKP2 expression and the promotion of proliferation are the result of cooperation between the signalization of growth factors and mechanical forces which affect a cell. In the studies carried out on smooth muscles and fibroblasts, it was shown that the growth factors regulate the level of SKP2 on the level of protein stabilization; in contrast, the increase of mechanical tension of cells causes the increase of protein expression on the level of transcription. Cell adhesion to substrate and mechanical tension of cells is conditional to maintain the transcription of SKP2 [102].

The activation of SKP2 promotor depends on a bind of transcription factor NFAT1 (nuclear factor of activated T cells). NFAT1 belongs to a family of four transcription factors which are activated by the level of calcium ions in the cytoplasm. Calcium ions, through the mechanism dependent on calmodulin, activate the phosphatase of calcineurin. Dephosphorylation of serine in NFAT induces conformational shifts which expose nuclear localization signal (NLS) and which cover the nuclear export signal (NES). A full dephosphorylation of NFAT1 leads to conformational shifts, which activates such protein functions as translocation to the nucleus, binding with the DNA and activation of transcription [103].

The transcription factor NFAT1 is activated in response to the increase of mechanical tension of a cell, which leads to the increase of expression of SKP2 protein. Conformational shifts of NFTA1, as a result of dephosphorylation, are tightly related to cells adhesion and the formation of mechanical forces dependent on the cell adhesion surface. This point supports the studies which show that the level of mRNA SKP2 in adherent cells may be regulated by the change in their shape. Results of many studies point that Skp2 is regulated by the influence of mechanical forces onto a cell. Shifts in mechanical tension of cells regulate mRNA level in bladder and vascular smooth muscle cells and skin fibroblasts. This leads to the assumption that the regulation of SKP2 transcription through actions of mechanical forces is an element of many, if not all, physiological and pathological processes dependent on the regulation of intensification of cell divisions, such as morphogenesis, tissues regeneration, and wound healing [104].

## 4. Development Prospects and Clinical Implications

Basic research of cell and tissue mechanobiology and clinical studies point to the importance of mechanical forces in the process of skin regeneration and wound healing. More important questions to be answered is how these molecules in specific pathways interact with each other in response to mechanical force and what controls target gene activity and what mechanosensing perturbed in skin regenerations and wound healing.

The outcome of these studies is the development of new therapies which use mechanical forces that support proper healing. It may be observed that the development of therapies based on the use of mechanical forces, or of bandages with appropriate mechanical properties, prevents improper scarring.

The importance of mechanical signaling in scars formation points to the observation associated with the use of botulinum toxin type A in aesthetic medicine (used to treat local subcutaneous muscle paralysis). The observations noted decrease of scarring in the areas where botulinum toxin was used; these effects are attributed to the reduced wound tension during its remodeling. Early clinical studies also show that injection of botulinum toxin into the wound site reduces the formation of hypertrophic scars [105].

Wounds auxiliary therapy treatment which uses devices that generate negative pressure (NPWT—negative pressure vacuum-assisted closure technology) is an effective method that supports extensive and rapid healing of chronic wounds. Functioning of NPWT facilitates the approximation of wound edges and stabilizes the environment, which reduces edema and ascites, and also reduces micromechanical forces [106].

Another beneficial therapy, which is deemed as effective physical modality for soft tissue wounds and which probably induces mechanisms of mechanotransduction and immunomodulation, is high-energy acoustic waves (ESWT—extracorporeal shock wave therapy) [107]. Results of the current studies suggest there is strong evidence documenting that ESWT application is safe and effective for the treatment of different etiologically soft tissue wounds, both acute and chronic. Clinical efficiency of ESWT shows a wide range of positive results, such as completed wound closure and reepithelialization, enhanced tissue granulation, reduced necrotic fibrin tissue, improved blood flow perfusion and angiogenesis, reduced period of total wound treatment, and decreased necessity of antibiotic treatment [108].

It seems that the mechanism of NPWT or ESWT, functioning as a technique for supporting wound healing, is based on mechanotransduction, and further researches are focused on the assessment of the optimal therapeutic parameters and the use of additional materials supporting therapy. The results of the studies and the opinions of clinicians show the importance of the transduction of mechanical forces in the process of wound healing and scar formation. The growing importance of mechanotransduction in wound healing and scar formation will contribute, to a large measure, to designing, to new clinical therapies, and to surgical procedures.

A better understanding of mechanobiology will enable the design of biomaterials, with appropriate physical and chemical properties, which will be used to treat improperly healing wounds. In addition, it will allow developing devices which will precisely control the mechanics of the wound and individualizing the therapy depending on the type, size, and anatomical location of the wound in certain patients, which will increase the efficiency of clinical therapy. Linking mechanobiology with the science of biomaterials and nanotechnology will enable in the near future a precise interference in abnormal cell signaling responsible for the proliferation, differentiation and cell death, and the restoration of biological balance.

In addition, knowledge of the mechanisms of mechanical signal transduction and its involvement in the activation of certain genes opens up new ways for combination therapies that use mechanical and drug therapy. This can increase the effectiveness of treatment.
